# Odontogenic deep neck space infection in a patient with hyper-IgE syndrome: A case report

**DOI:** 10.4317/jced.55239

**Published:** 2018-10-01

**Authors:** Tsutomu Sugiura, Kazuhiko Yamamoto, Kazuhiro Murakami, Tadaaki Kirita

**Affiliations:** 1 DDS, PhD, Clinical Associate Professor, Department of Oral and Maxillofacial Surgery, Nara Medical University; Director, Department of Oral and Maxillofacial Surgery, Nara Kasuga Hospital, Nara, Japan; 2 DDS, PhD, Clinical Professor, Department of Oral and Maxillofacial Surgery, Nara Medical University, Nara, Japan; 3 DDS, PhD, Clinical Instructor, Department of Oral and Maxillofacial Surgery, Nara Medical University, Nara, Japan; 4 DDS, DMSc, Professor and Chair, Department of Oral and Maxillofacial Surgery, Nara Medical University, Nara, Japan

## Abstract

Hyperimmunoglobulin E syndrome is a primary immunodeficiency state that is characterized by eczema, recurrent skin and lung infections, and markedly increased levels of IgE. Odontogenic infection can spread to vital and deep structures in such immunocompromised patients. We report a case of a 19-year-old man with hyperimmunoglobulin E syndrome presenting deep neck space infection that had spread from periapical periodontitis of the lower molars. A computed tomography scan showed an area of bony destruction in the left mandible and abscess formation in the submandibular and submental spaces. The patient was successfully treated by cervical drainage, extraction of the causative teeth, and antibiotic therapy. The present case highlights the importance of adequate treatment of dental infections in immunocompromised patients.

** Key words:**Hyperimmunoglobulin E syndrome, odontogenic infection.

## Introduction

Hyperimmunoglobulin E syndrome (HIES) is a rare multisystem immunodeficiency disease characterized by high serum immunoglobulin E (IgE) levels, eczema, and recurrent infections. Common findings include significant atopic eczema, staphylococcal dermatitis, cellulitis and folliculitis, parenchymal lung disease, osteopenia, and frequent long bone fracture (1). Oral manifestations such as delayed eruption of the permanent teeth, a high arch palate, and recurrent oral candidiasis are also common (2). Because of the immune deficiency of HIES, odontogenic infection can quickly become complicated and severe (3,4). However, there have been only a few reports on the management of odontogenic infections in a patient with hyper-IgE syndrome. This report describes the management of a case of hyper-IgE syndrome complicated by a deep neck space infection caused by periapical periodontitis of the lower molars.

## Case Report

A 19-year-old man was referred to the Department of Oral and Maxillofacial Surgery at Nara Kasuga Hospital with a complaint of swelling at the left submandibular region. He had felt pain and swelling at the left molar region several times in the previous 2 years. He consulted his internist at the Department of Infectious Disease at Nara City Hospital and was administered antibiotics and analgesics. The patient was strongly recommended to undergo dental treatment. However, he refused to receive dental treatment due to dental anxiety. When the pain and swelling developed, he presented at otolaryngology department at Nara City Hospital and was treated with intraoral incision and drainage.

The patient was diagnosed with HIES at 1 month of age and had been followed-up by the Department of Pediatrics until the age of 18. Genetic analysis for signal transducer and activator of transcription 3 (STAT3) mutation was positive. He had multiple episodes of subcutaneous abscess on his lower legs and buttocks, perianal abscess, recurrent pneumonia, and osteomyelitis of the foot. Cultures of his abscesses revealed methicillin-resistant Staphylococcus aureus in most cases. These infections usually became severe and showed slow improvement. Delayed primary tooth shedding was noted by his dentist. The patient’s medications included minocycline hydrochloride (200 mg/day), levocetirizine dihydrochloride (5 mg/day), domperidone (30 mg/day), and amphotericin B.

**Figure 1:**
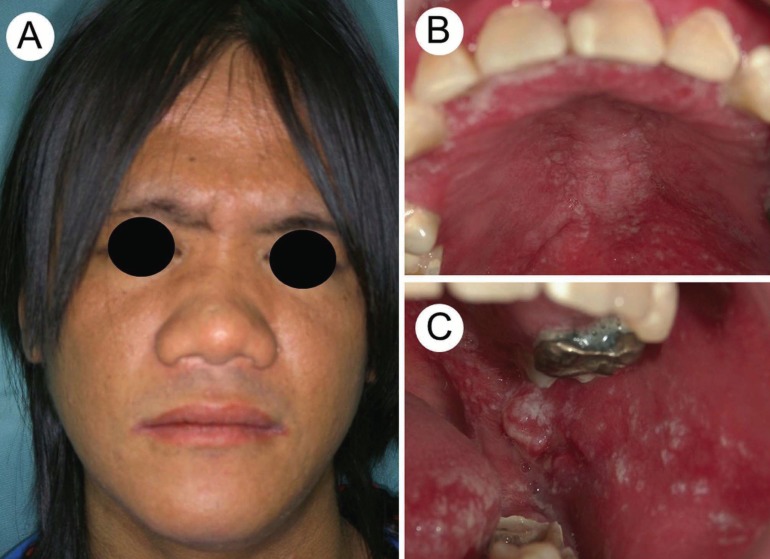
Clinical view at the first visit. (A) The patient’s face showing a prominent front and broad nose base. (B) Candidiasis of the palate and multiple dental caries. (C) Candidiasis of the left buccal mucosa and tongue.

**Figure 2:**
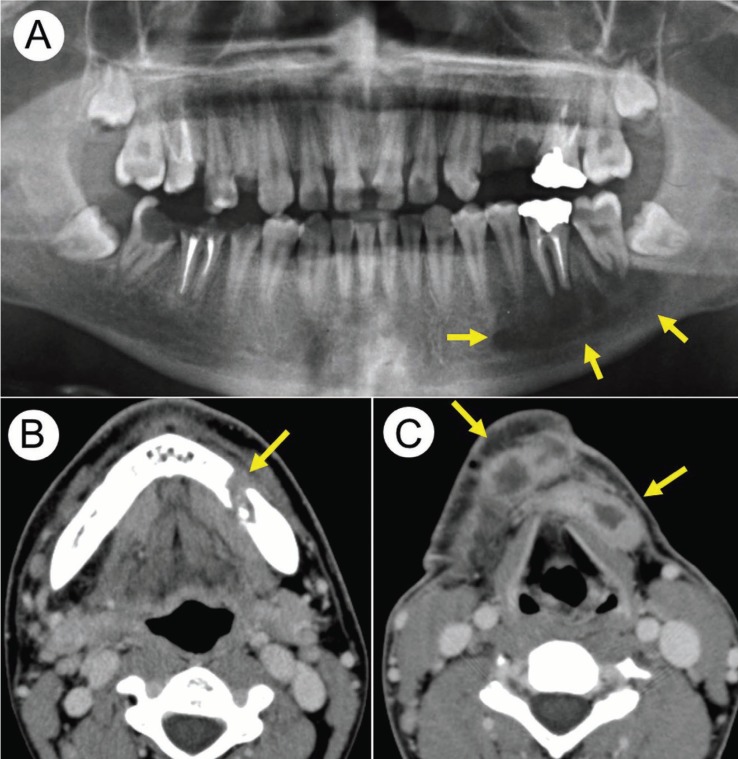
(A) Panoramic radiograph at the first visit (6 months preoperatively). (B) and (C) CT scan 1 week before cervical drainage and necrotomy.

On physical examination, the patient’s body size was smaller than normal for his age and sex. His face was coarse, with a prominent forehead and broad nasal bridge. Diffuse swelling was observed in the left submandibular area. Oral examination disclosed gingival swelling, candidiasis at the palatal and buccal mucosa, and multiple dental caries (Fig. 1). A panoramic radiograph revealed a large periapical bone resorption involving the lower left first and second molars (Fig. 2A). The clinical diagnosis was perimandibular abscess due to periapical periodontitis of the left lower first and second molars. He underwent root canal treatment several times; however, he stopped visiting our hospital.

**Figure 3:**
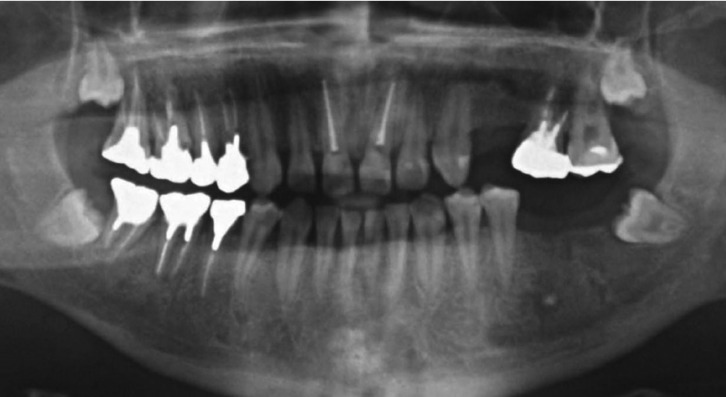
Panoramic radiograph 7 months after surgery.

The patient felt submandibular swelling and dysphagia 5 months after his last visit to our hospital and consulted his otolaryngologist. Swelling was observed extending from the left submandibular region to the submental area. A computed tomography scan showed an area of bony destruction in the left mandible and abscess formation in the submandibular and submental spaces (Fig. 2B,C). Laboratory examination showed an infection, with a white blood cell count of 13,000/mm3 and C-reactive protein of 8.0 mg/dL. His previous laboratory reports were unremarkable except for significantly increased IgE levels (4000-8000 IU/mL). Under a diagnosis of submandibular and submental abscess, the patient was admitted to the Otolaryngology Department of Nara City Hospital. Drainage and necrotomy of the neck abscess was performed under general anesthesia by otolaryngologists. Microbiological culture yielded Peptostreptococcus spp., Escherichia coli, and Clostridium spp. Intravenous administration of sulbactam/ampicillin 3 g 4 times daily was provided for 3 days. C-reactive protein markedly decreased to 0.98 mg/dL 3 days after drainage and necrotomy. Four days after drainage and necrotomy, the patient was referred to our hospital. The left first and second lower molars were extracted and the periapical lesion was curetted under intravenous sedation with midazolam by the oral and maxillofacial surgeon. Additional intravenous administration of sulbactam/ampicillin 3 g 4 times daily was provided for 3 days, followed by oral administration of amoxicillin hydrate (3 g) and amoxicillin-clavulanic acid (3 g) for 30 days, with reference to the dose and duration of antibiotics for the treatment of previous subcutaneous abscesses. The patient was transferred to our hospital and admitted to the Department of Oral and Maxillofacial Surgery 7 days after drainage and necrotomy. The treatment of multiple dental caries and periodontitis was done under intravenous sedation 3 times per week. Postoperative healing was uneventful and the patient was discharged 4 weeks after surgery. He continued to undergo restorative and prosthodontic treatment. At 7 months after surgery, he showed no signs of intraoral infection. A panoramic radiograph showed bone regeneration at the left posterior mandible and no periodontal lesions of the teeth (Fig. 3).

## Discussion

Hyper-IgE syndrome is a rare primary immunodeficiency disorder that can be characterized by eczema, recurrent skin and lung infections, skeletal abnormalities, and extremely elevated serum IgE, with an incidence of less than 1 case per million (1). It was first described by Davis *et al.* in 1966 before the discovery of IgE and was called Job’s syndrome because of “cold abscess” of the skin (5). The syndrome was further delineated by Buckley *et al.* with the recognition of extremely high serum IgE levels (6). There are two forms of HIES: an autosomal dominant form (AD-HIES) and a recessive form (AR-HIES). Recently, the cause of HIES was identified as a genetic mutation of STAT3 and TYK2 (tyrosine kinase 2) (7). STAT3 is a major signal transduction protein required for normal wound healing, angiogenesis, immune pathways, and protection against cancer. The pathophysiology of HIES is not yet completely understood; however, loss-of-function STAT3 mutations result in HIES. The AR-HIES form is probably ascribable to either a mutation of the TYK2 gene or the dedicator of cytokinesis 8 (DOCK8) (2).

The clinical features of HIES include the immune system, connective tissue, skeleton, and dental development with variations in the severity of the symptoms. Recurrent skin abscess, pneumonia with pneumatocele formation, mucocutaneous candidiasis, elevated serum IgE, and eosinophilia are the most common features of immunodeficiency and immune dysregulation in HIES (1). Frequent pathogens isolated in HIES are Staphylococcus aureus, Streptococcus pneumoniae, Haemophilus influenzae, and Candida that typically respond promptly to appropriate antimicrobial therapy. Compared to the AD-HIES, the AR form lacks the somatic features, such as the typical faces, scoliosis, and dental alterations (1,8). The present case was compatible with AD-HIES.

Common facial characteristics include facial asymmetry, deep-set eyes, increased alar and outer canthal distance, a broad nasal bridge, and a prominent forehead. These were noticed in the present case. Delayed primary tooth shedding is also a common feature of HIES. Reduced resorption of the primary tooth root is thought to lead to prolonged retention of the primary teeth, which in turn prevents the appropriate eruption of the permanent teeth (9).

Previously, the diagnosis was based solely on the clinical and laboratory features. Since the discovery of STAT3 mutations, the diagnosis may be based on a high level of clinical suspicion and confirmed through STAT3 mutational analysis (7). Although STAT3 is the only gene thus far identified to cause autosomal dominant HIES, it remains possible that more than one genetic etiology exists.

As there is no cure for HIES, treatment is based on prophylactic antimicrobials and skin care. The frequency and length of antibiotic prophylaxis depends on medical decision because no controlled trials have been conducted. Systemic bacterial or fungal infections are often severe and a selection of the correct antibiotic drug is crucial. Amoxicillin or amoxicillin-clavulanic acid is considered the option of first choice to treat odontogenic infection (10). In this case, considering a large infected lesion with bone destruction around the first and second lower molars, we administered antibiotics for a month postoperatively.

Of primary concern to dental surgeons are immunologic disturbances that quickly change a localized odontogenic infection into a severe infection in a short period. Only a few case of HIES have been reported in the dental literature. A literature search of PubMed using the keywords “hyper-IgE syndrome,” “dental (or oral),” and “case report” from 1983 to 2017 revealed 11 articles including 16 cases of HIES managed by dentists in the English literature (11-15). Of these, there were only 2 articles on odontogenic infections: a case of life-threatening deep space neck infection due to a periapical abscess (4) and prolonged cellulitis of the floor of the mouth arising from a periapical infection of a molar (3). These patients exhibited abnormally clinical improvement after treatment. In the present case, the lack of initial dental treatment due to the patient’s dental anxiety was another reason why the periapical lesion widely spread to the submandibular and submental areas. The present case highlights the importance of oral health maintenance and adequate treatment of dental infections in patients with HIES.
